# The clinical significance, natural history and predictors of bone marrow lesion change over eight years

**DOI:** 10.1186/ar4611

**Published:** 2014-07-14

**Authors:** Yi Chao Foong, Hussain Ijaz Khan, Leigh Blizzard, Changhai Ding, Flavia Cicuttini, Graeme Jones, Dawn Aitken

**Affiliations:** 1 Menzies Research Institute Tasmania, University of Tasmania, Private Bag 23, Hobart, Tasmania 7000, Australia; 2 Department of Epidemiology and Preventive Medicine, School of Public Health and Preventive Medicine, Monash University, Alfred Hospital, Melbourne, Victoria, Australia

## Abstract

**Introduction:**

There is increasing evidence to suggest that bone marrow lesions (BMLs) play a key role in the pathogenesis of osteoarthritis (OA). However, there is a lack of long term data. The aim of this study was to describe the natural history of knee BMLs, their association with knee pain and examine predictors of BML change over eight years.

**Methods:**

A total of 198 subjects (109 adult offspring of subjects who had a knee replacement and 89 community-based controls) were studied. Knee pain and BML size were assessed at two and ten year visits.

**Results:**

At the two year visit, 64% of participants (n = 127) had 229 BMLs (34% patella, 26% femoral and 40% tibial). Over eight years, 24% (55/229) increased in size, 55% (125/229) remained stable and 21% (49/229) decreased in size or resolved completely. Of the participants without BMLs at baseline, 52% (37/71) developed incident BMLs.

After adjusting for confounders, eight year change in total BML size was associated with change in knee pain in offspring (β = 2.50, 95% confidence interval (CI) 0.96 to 4.05) but not controls. This association was stronger in males. Incident BMLs were associated with increase in pain (β = 3.60, 95% CI 1.14 to 6.05). Body mass index (BMI) and strenuous activity (but not radiographic osteoarthritis or smoking) were associated with an increase in BML size.

**Conclusion:**

In this midlife cohort, the proportion of BMLs increasing in size was similar to those decreasing in size with the majority remaining stable. Change in BMLs was predicted by BMI and strenuous activity. An increase in BML size or a new BML resulted in an increase in pain especially in males and those with a family history of OA.

## Introduction

Osteoarthritis (OA) is the most common joint disorder worldwide, and the knee is the most common joint affected [1,2]. Bone marrow lesions (BMLs) play a key role in the pathogenesis of knee OA – they are associated with OA symptoms such as pain and function, and predict cartilage loss and joint replacement surgery [3-6]. In a recent Delphi exercise that aimed to establish a definition for OA on magnetic resonance imaging (MRI), BMLs were included as a key component of the diagnostic criteria [7].

The current literature on the natural history of BMLs is conflicting, with significant variation depending on the study population. A study in a healthy population has shown that incident BMLs developed in 14% of individuals over 2 years, and that knee pain was more likely to develop in these participants [8]. The same study showed that nearly one-half of the BMLs present at baseline completely resolved, while another study of middle-aged healthy women over 2 years found similar results [9]. Studies in symptomatic OA populations generally show a lower percentage of BMLs resolving, with one study reporting that less than 1% of patients showed a BML decrease over 30 months [10]. Other studies have quoted higher figures, with 10% of BMLs resolving over 2 years in a study by Kornaat and colleagues [11]. Our group has previously reported that rates of incident BMLs were low (7%), with about one-quarter of BMLs showing an increase or a decrease in size over 2.7 years in a population-based cohort of older adults with and without OA [3]. The reasons behind these variations are unclear; however, it is worth noting that no study has looked at the natural history of BMLs beyond 3 years.

Pain is a key criterion for a clinical diagnosis of OA. A number of studies have reported an association between BMLs and pain across a range of demographics and activities [12-16]. Furthermore, longitudinal studies have shown that increases in BML size and incident BMLs are both associated with increasing knee pain over 2 to 3 years [17,18]. However, some studies have shown no association between BMLs and pain longitudinally [11,19] or cross-sectionally [20].

Given the role of BMLs in OA, there has been interest in the risk factors that lead to an increased risk of developing BMLs. There is significant overlap with the major risk factors for OA, and age and weight have been shown to be some of the strongest risk factors for BMLs [21,22]. Physical activity, particularly doing over 10,000 steps per day, may aggravate existing BMLs [23]. Recently, vascular risk factors have also been implicated due to their effects on blood flow in the small vessels of subchondral bone [24]. Smoking, increased serum glucose levels, serum cholesterol and triglyceride, fatty acid intake, carbohydrate intake and changes in retinal microvasculature have all been associated with BMLs [25-29]. There is also a significant genetic component [30].

The conflicting data on natural history and clinical significance may be attributable to the differing methodology in many of these studies. These include differences in imaging protocols, sample size, age, sites measured, and severity of OA in the study sample. Importantly, few studies have followed the progression of BMLs beyond 3 years. The aims of this study were to describe the natural history of BMLs over 8 years, to examine the relationship between change in BML size and change in knee pain, and to examine factors predicting change in BML size.

## Materials and methods

### Study subjects

This study was conducted as part of the Offspring study, which is an ongoing population-based study. The Offspring study began in southern Tasmania (primarily in the capital city of Hobart) in June 2000. One-half of the participants were the adult offspring of patients who had a knee replacement performed for idiopathic knee OA at any Hobart hospital from 1996 to 2000 [31]. The diagnosis was confirmed by reference to the medical records of the orthopedic surgeon and the original radiograph when possible. Controls were age and sex matched and were randomly selected from the population. Participants were excluded if they had a contraindication to MRI (including metal sutures, presence of shrapnel, iron filing in eye, or claustrophobia). This study includes data from the second and third visits at approximately 2 and 10 years respectively, because T2-weighted MRI scans were not performed at baseline.

The Southern Tasmanian Health and Medical Human Research Ethics Committee approved the protocol, and written informed consent was obtained from all participants.

### Anthropometrics

Weight was measured to the nearest 0.1 kg (with the subject’s shoes, socks, and bulky clothing removed), with a single pair of electronic scales (Delta Model 707; Seca, Munich, Germany) that were calibrated using a known weight at the beginning of each clinic session. Height was measured to the nearest 0.1 cm (with shoes and socks removed) using a stadiometer. Body mass index (BMI) was calculated as weight (kg)/height (m^2^). Smoking was assessed by questionnaire and categorized as current smoker, past smoker or never smoked [32]. Physical activity was also assessed by a self-administered questionnaire that assessed the amount of time spent in light and strenuous physical activity on a five-point scale [33]. Participants were asked about the number of days during the last 14 days spent doing at least 20 minutes of strenuous exercise (that is, bicycling, brisk walking, jogging, aerobics, and so forth that was enough to raise your pulse rate or cause you to breathe faster) and light exercise (that is, walking, light housework, slow bicycling, and so forth that was not severe enough to cause a pulse rate rising or breathing increase). The participants then chose a score between 1 and 5, where score 1 represents no days, score 2 represents 1 or 2 days, score 3 represents 3 or 5 days, score 4 represents 6 or 8 days, and score 5 represents 9 days or more of exercise.

### Leg strength

Leg strength was measured by dynamometry at the lower limb, involving both legs simultaneously. This primarily involves the hip flexors and knee extensors. Each measure was performed twice, with instructions given prior to testing. The repeatability estimate (Cronbach’s alpha) was 0.91. The device was calibrated by suspending known weights at regular intervals [34].

### Knee pain

Knee pain was assessed by self-administered questionnaire using the Western Ontario and McMaster Universities Osteoarthritis Index at both visits [35]. Five categories of pain (walking on flat surface, going up or down stairs, at night, sitting or lying, and standing upright) were assessed separately with a 10-point scale from 0 (no pain) to 9 (most severe pain). Each score was then summed to create a total pain score (range 0 to 45).

### Radiography

A standing anteroposterior semiflexed view of the right knee (at 15° flexion) was performed in all participants at baseline and 10 years. Radiographs were scored individually for osteophytes and joint space narrowing. Each of the following four features was scored on a scale from 0 to 3 (0 = normal and 3 = severe): medial joint space narrowing, lateral joint space narrowing, medial osteophytes (femoral and tibial combined), and lateral osteophytes (femoral and tibial combined). Each score was arrived at by consensus with two readers simultaneously assessing the radiograph with immediate reference to the Osteoarthritis Research Society International atlas [36]. A nonzero score in either joint space narrowing or osteophytosis was regarded as evidence of radiographic osteoarthritis (ROA). Reproducibility was assessed in 50 radiographs, 2 weeks apart, and yielded an intraclass correlation coefficient of 0.99 for osteophytes and 0.98 for joint space narrowing.

### Magnetic resonance imaging

An MRI scan of the right knee was performed on a 1.5 T whole-body magnetic resonance unit (Picker, Cleveland, OH, USA) with the use of a commercial transmit–receive extremity coil. Knees were imaged in the sagittal plane and the following image sequences were used: visit two, a T2-weighted fat saturation two-dimensional fast spin echo (flip angle 90°; repetition time 3,067 ms; echo time 112 ms; field of view 16 cm; 256 × 256 matrix; slice thickness of 4 mm with a between-slices gap of 0.5 to 1.0 mm); and visit three, a T2-weighted fat saturation two-dimensional fast spin echo (flip angle 90°; repetition time 3,067 ms; echo time 112 ms; field of view 16 cm; 256 × 256 matrix; slice thickness of 2 mm with a between-slices gap of 0.5 mm).

Visit one only involved T1 MRI scans, which were not suitable for comparison of BMLs over time. Subchondral BMLs were assessed using Osirix software (University of Geneva, Geneva, Switzerland) and were defined as areas of increased signal adjacent to the subcortical bone at the medial tibial, medial femoral, lateral tibial, lateral femoral, superior patella, and inferior patella sites as described previously [3]. One trained observer scored the BMLs by measuring the maximum area (cm^2^) of the lesion at both time points. The observer manually selected the MRI slice with the greatest BML size. The BML with the largest size was recorded if more than one lesion was present at the same site. MRIs at both time points were read paired with the chronological order known to the observer but blinded to clinical status. Participants were given a BML score (cm^2^) for each of the six sites (medial tibial, medial femoral, lateral tibial, lateral femoral, superior patella, and inferior patella sites) as well as a total BML score, which was the sum of the scores at each site. Change in BML size was then calculated by subtracting the visit two BML size from the visit three BML size. Intraobserver repeatability was assessed in 40 subjects with at least a 2-week interval between the readings. The intraclass correlation coefficient was 0.97.

To examine the natural history of BMLs, a significant change in BML size was defined as any change above or below the least significant criterion (LSC). The LSC takes into account measurement error and the correlation between BML measurements at baseline and follow-up. The formula is as follows, where *σ* is the standard error of the mean and *ρ* is the serial correlation:

$$\mathrm{LSC}=1.96\times \sigma \sqrt{2\left(1-\rho \right)}$$

The LSC was calculated for each of the six sites in the knee (11 mm^2^ for medial femoral, 17 mm^2^ for lateral femoral, 16 mm^2^ for medial tibial, 14 mm^2^ for lateral tibial, 15 mm^2^ for superior patellar, and 13 mm^2^ for inferior patellar BMLs). This was then used to calculate the number of BMLs increasing and decreasing in size, where an increase in BML size was defined as any change greater than the LSC, and *vice versa* for a decrease in BML.

Meniscal damage was assessed by a trained observer on T1-weighted MRI scans as described previously [37]. Each meniscus is divided into three segments (anterior horn, body and posterior horn) for the assessment of both meniscal extrusions and tears.

Extrusion is defined as when meniscal tissue extends beyond the tibial margin, and complete extrusion is defined as when the meniscus has no contact with the joint space. For extrusions, each segment (anterior horn, body, and posterior horn) of both medial and lateral menisci were scored on a scale from 0 to 2 (0  =  no extrusion, 1  =  partial meniscal extrusion, 2  =  complete meniscal extrusion with no contact with the joint space). Each meniscus can have a maximum score of 6 and a total knee score of 12 for extrusions.

A maximum score of 6 can be given for tears (0  =  no damage, 1  =  one of three meniscal areas involved (anterior, middle, and posterior horns), 2  =  two of three areas involved, 3  =  all three areas involved). This value was then scored for both medial and lateral menisci, giving a total score of 6.

These scores were summed to create a total meniscal pathology score, which had a possible range from 0 to 18.

### Statistical analysis

The characteristics of study participants were compared using an independent-samples *t* test for continuous variables and a chi-squared test for categorical variables. Linear regression was used to estimate the relationship between change in pain and change in BML size. This was performed using total BML area (summed across all six sites) as well BML area at each site specifically. Multivariable analyses were adjusted for age, sex, BMI, leg strength, and the presence of ROA. Interactions between BML change and sex, and between BML change and offspring–control status, were assessed from the coefficient and its standard error of product terms was formed from the covariates for the study factors involved. Linear regression was also used to examine potential factors predicting a change in BML size. Univariable analysis was performed with a range of lifestyle and demographic factors (BMI, physical activity, smoking status, ROA, and offspring–control status) and those that were significantly associated were included in the multivariable model together with covariates for age and sex to adjust for these factors.

Standard diagnostic checks of model adequacy were performed on all final models. Residuals from all models were normally distributed, or approximately so, without evidence of heteroskedasticity.

Values of *P* <0.05 were considered statistically significant. All statistical analysis was performed on Intercooled Stata 12.0 for Windows (StataCorp LP).

## Results

### Participant characteristics

The participants in this study were 198 subjects with complete MRI measures at the 2-year and 10-year visits (52.7% of those studied at baseline). There were no significant differences in sex, BMI, age, height, weight, frequency of ROA, and pain at baseline between those lost to follow up (*n* = 178) and the participants in our study (*n* = 198) (data not shown). Table 1 presents the characteristics of the study sample. BMLs were present in 64% (127/198) of the sample at visit two, with an average BML size of 0.63 cm^2^. Of note, those with BMLs reported higher levels of pain at visit two and had a greater change in BML size. There were no significant differences in sex, age, BMI, offspring status, physical activity levels, height, weight, or change in pain, although those with BMLs tended to have a higher proportion with ROA compared with those with no BMLs. The mean (standard deviation) of physical activity in our cohort was 2.61 (1.33) for strenuous and 4.09 (1.11) for light activity respectively (data not shown).

**Table 1:** Participant characteristics

	**BML present (*n*** **= 127)**	**BML not present (*n*** **= 71)**	***P *** **value**
Females (%)	41% (51/127)	47% (33/71)	0.4
Age (years)	47.6 (6.5)	47.2 (6.0)	0.65
Offspring (%)	58% (74/127)	49% (35/71)	0.22
BMI (kg/m^2^)	28.0 (5.8)	27.0 (4.2)	0.24
Height (cm)	168.5 (9.2)	169.1 (8.9)	0.7
Weight (kg)	79.6 (18.1)	77.6 (15.1)	0.43
Leg strength (kg)	114.9 (47.4)	118.3 (44.3)	0.64
Active smokers (%)	17.3	15.5	0.77
Light activity (per unit change)	4.1 (0.09)	4.00 (0.15)	0.43
Strenuous activity (per unit change)	2.65 (0.12)	2.52 (0.15)	0.51
Radiographic osteoarthritis (%)	20% (26/127)	10 (7/71)	0.06
Pain score (0 to 29)	**3.7 (5.9)**	**1.3 (3.1)**	**<0.01**
Change in pain score (-25 to 44)	2.5 (8.1)	2.2 (4.2)	0.84
Total BML area (0 to 4.10 cm^2^)	**0.63 (0.80)**	**–**	**–**
MF BML area (0 to 1.98 cm^2^)	**0.37 (0.50)**	**–**	**–**
LF BML area (0 to 2.54 cm^2^)	**0.64 (0.75)**	**–**	**–**
MT BML area (0 to 1.83 cm^2^)	**0.33 (0.36)**	**–**	**–**
LT BML area (0 to 1.99 cm^2^)	**0.26 (0.31)**	**–**	**–**
SP BML area (0 to 1.70 cm^2^)	**0.38 (0.34)**	**–**	**–**
IP BML area (0 to 1.06 cm^2^)	**0.23 (0.27)**	**–**	**–**
Change in BML area (-2.09 to 4.46 cm^2^)	**0.61 (1.03)**	**0.28 (0.46)**	**0.01**

Values were taken from visit two of the study, except for radiographic osteoarthritis that was taken from visit one. Change refers to the difference in values between visit two and visit three. Values represent mean (standard deviation) unless percentages. Light and strenuous activity were rated on a five-point scale: 1 = no days, 2 = 1 or 2 days, 3 = 3 to 5 days, 4 = 6 to 8 days, and 5 = 9 days or more spent doing at least 20 minutes of the respective level of activity in the past 14 days. BMI, body mass index; BML, bone marrow lesion; IP, inferior patella; LF, lateral femoral; LT, lateral tibial; MF, medial femoral; MT, medial tibial; SP, superior patella. Bold data indicate *P* <0.05.

### Natural history

**Figure 1:**
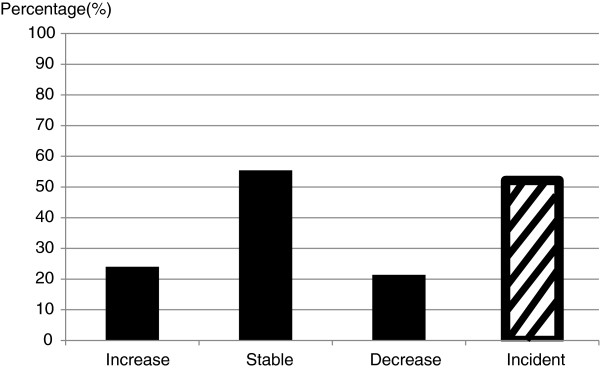
Natural history of bone marrow lesions.

The 127 participants with a BML had a total of 229 BMLs present at visit two (58 had a BML at one site, 45 had a BML at two sites, 17 had a BML at three sites, five had a BML at four sites and two had a BML at five sites). Figure 1 describes the natural history of these BMLs. Roughly one-quarter of BMLs increased (*n* = 55) or decreased (*n* = 49) in size, whilst the remainder remained unchanged in size (*n* = 125) based on a change less than the LSC. Of those without BMLs at baseline (*n* = 71), slightly over one-half developed one or more incident BMLs (*n* = 37) over the 8 years. There was no significant difference in natural history between offspring and controls (data not shown).

### Pain

Table 2 presents associations between change in total BML size and change in pain. Every 1 cm^2^ increase in BML size resulted in a 1.53 (95% confidence interval = 0.37, 2.70) unit increase in pain score after adjustment for age, sex, BMI, leg strength, and the presence of ROA. Adjusting for baseline joint space narrowing, osteophytes, meniscal extrusion, and meniscal tears did not significantly affect our findings (<10% change in the beta coefficient following further adjustment). A significant offspring–control interaction was present (*P* = 0.08), with change in BML size more strongly associated with change in pain among offspring than controls. Furthermore, this association was stronger in males compared with females among both the offspring group and the whole sample.

**Table 2:** Relationship between change in WOMAC and change in BML size

	**Univariable**	**Multivariable**^**a**^	**Females**^**a**^	**Males**^**a**^
Total	**1.74 (0.65, 2.84)**	**1.53 (0.37, 2.70)**	0.63 (-0.93, 2.20)	**2.53 (0.76, 4.30)**
Offspring	**2.68 (1.22, 4.13)**	**2.50 (0.96, 4.05)**	**2.25 (0.10, 4.41)**	**3.06 (0.88, 5.24)**
Controls	-0.05 (-1.72, 1.62)	-0.39 (-2.23, 1.44)	-0.77 (-3.51, 1.98)	1.44 (-1.25, 4.14)

Data presented as beta coefficient (95% confidence interval). Values are the change in WOMAC pain score per cm^2^ change in BML size. An interaction between offspring–control status and change in BML size (*P* = 0.01) as well as sex and change in BML size (*P* = 0.08) on change in pain was present. BML, bone marrow lesion; WOMAC, Western Ontario and McMasters Universities Arthritis Index. Bold data indicate *P* <0.05. ^a^Adjusted for age, sex, body mass index, leg strength, and radiographic osteoarthritis.

Table 3 presents associations between site-specific change in BML size and change in pain. Changes in medial and lateral tibial BMLs were significantly associated with change in pain, with the association stronger among offspring at the medial tibial site (*P* value for interaction was <0.01). After adjustment for age, sex, BMI, leg strength, and ROA, change in lateral tibial BMLs and change in pain was no longer significantly associated. No significant association was found between change in BML size and change in pain at other sites.

**Table 3:** Relationship between change in WOMAC and site-specific change in BML size

	**Univariable**	**Multivariable**^**a**^
Medial tibial	**2.96 (0.59, 5.34)**	**3.67 (0.89, 6.45)**
Controls	-1.39 (-4.18, 1.40)	-3.38 (-7.15, 0.39)
Offspring	**8.99 (5.30, 12.68)**	**8.98 (5.22, 12.73)**
Lateral tibial	**2.37 (0.08, 4.66)**	1.98 (-0.36, 4.32)
Medial femoral	2.11 (-1.14,5.36)	1.63 (-1.64, 4.90)
Lateral femoral	-0.09 (-2.41, 2.24)	-0.69 (-3.11, 1.73)
Superior patella	0.74 (-2.73, 4.21)	0.61 (-2.85, 4.08)
Inferior patella	3.95 (-0.59, 8.50)	4.30 (-0.26, 8.86)

Data presented as beta coefficient (95% confidence interval). Values are the change in WOMAC pain score per cm^2^ change in BML size. An interaction between offspring and control status was present at the medial tibial site (*P* <0.01). BML, bone marrow lesion; WOMAC, Western Ontario and McMasters Universities Arthritis Index. Bold data indicate *P* <0.05. ^a^Adjusted for age, sex, body mass index, leg strength, and radiographic osteoarthritis.

For those with no pain or BMLs present at baseline (*n* = 42), the development of a BML was significantly associated with an increase in pain after adjustment for age, sex, BMI, leg strength, and ROA in offspring and controls combined with a change in pain score of 3.60 (95% confidence interval = 1.14 to 6.05) points per 1 cm^2^ change in BML size (not shown in Table 3).

### Factors affecting bone marrow lesion change

Table 4 presents predictors of BML change. BMI and strenuous activity were deleteriously associated with change in BML size. These associations remained statistically significant after adjusting for age and sex and each other. Smoking status, the presence of ROA, light activity, offspring–control status, and leg strength was not associated with change in BML size.

**Table 4:** Predictors of change in bone marrow lesion size

	**Univariable**	**Multivariable**^**a**^
Body mass index (kg/m^2^)	**0.03 (0.00, 0.05)**	**0.03 (0.01, 0.05)**
Strenuous activity (per unit change)	**0.13 (0.03, 0.22)**	**0.14 (0.04, 0.23)**
Current smoker (yes/no)	0.04 (-0.13, 0.20)	–
Ever smoker (yes/no)	0.06 (-0.20, 0.31)	–
Radiographic osteoarthritis (yes/no)	0.28 (-0.06, 0.61)	–
Light activity (per unit change)	0.02 (-0.10, 0.13)	–
Offspring status (yes/no)	0.13 (-0.11, 0.38)	–
Leg strength (kg)	0.00 (0.00, 0.00)	–

Data presented as beta coefficient (95% confidence interval). Values are the change in bone marrow lesion size (cm^2^) per unit change in covariates. All factors are from visit two, except for radiographic osteoarthritis that was collected at visit one. Strenuous and light activity were assessed on a five-point scale: 1 = no days, 2 = 1 or 2 days, 3 = 3 to 5 days, 4 = 6 to 8 days and 5 = 9 days or more. Bold data indicate *P* <0.05. ^a^Adjusted for age, sex, body mass index, and strenuous activity.

## Discussion

This population-based study of middle-aged adults has investigated the natural history of BMLs over 8 years and the association between change in BML size and change in pain. Incident BMLs were common; roughly one-half of those without BMLs at visit two developed new BMLs by visit three. Of the BMLs present at visit two, 55% remained stable while 24% increased and 21% decreased in size. An increase in BML size or new BML resulted in a significant increase in knee pain, especially for male offspring. BMI and strenuous activity independently predicted change in BML size.

This is the first study to report the natural history of BMLs over an extended period of time. Many of the previous studies have been conducted over a much shorter timeframe. Davies-Tuck and colleagues reported a much higher proportion of BMLs improving, with 46% of BMLs resolving completely in a healthy, pain-free population over 2 years [8]. In a symptomatic population with ROA, less than 1% of BMLs resolved or reduced in size [10]. The conflicting data may be a reflection of different study populations as well as different grading systems used for the assessment of BMLs. In this study, patellar BMLs were also assessed, which may explain the high percentage of participants with BMLs compared with other studies that did not assess patella BMLs [3,8,9]. A previous study by our group in a population-based sample of older adults that employed the same quantitative BML methodology found very similar results to our current study where approximately one-quarter of BMLs both increased and decreased in size [3].

In our study, incident BMLs in subjects without BMLs at visit two were also high, most probably due to the period of follow-up. Other studies have reported much lower figures, ranging from 9 to 14% in a healthy population over 2 years [8,9] and 20% in a cohort with symptomatic knee OA over 30 months [10]. Of clinical importance, the development of an incident BML was significantly associated with the development of pain in those who were pain free at visit two. This association has been corroborated by a prior study looking at healthy populations [8] and a cohort consisting of subjects with OA or at high risk of OA [17]. This observation further lends weight to the argument that BML development may be a major contributor to incident knee pain.

A 1 cm^2^ increase in BML size resulted in a 2.5 unit increase in knee pain in those with a family history of OA, whereas no association was seen in controls. Previous findings from this cohort have shown that offspring with a family history of knee replacement were more likely to have a greater BMI, more knee pain, and less muscle strength cross-sectionally compared with matched controls [31]. However, there have been few studies examining the genetic factors influencing BMLs. Zhai and colleagues reported that BMLs have a significant genetic component in this cohort [30], but they did not investigate whether there was a genetic component to the role that BMLs play in pain. BMLs are perhaps more likely to cause pain in genetically susceptible individuals given that genes can discriminate those with OA and pain from those with OA without pain [38]. Alternatively, BML pathology may be different in those with a family history of OA and MRI is somewhat nonspecific with regard to the underlying pathology.

The association between change in BMLs and change in pain was also stronger in males compared with females. Few studies have reported on sex difference in BMLs. Davies-Tuck and colleagues reported that sex was not associated with the presence, development or persistence of BMLs [8], whilst Dore and colleagues found that males were more likely to have BMLs and have a BML increase over time [3]. In our cohort, males had significantly larger BMLs at both visit two and visit three. When we adjusted our model for BML size at visit two and visit three, however, the sex difference persisted, suggesting that there may be a difference in the way BMLs mediate pain between sexes.

When we examined site-specific BMLs and their association with pain, we found that tibial BMLs were associated with change in pain but not patellar or femoral BMLs. In particular, medial tibial BMLs were strongly associated with change in pain for offspring. To the best of our knowledge, no study has looked at site-specific associations between BMLs and pain. Studies have shown that BMLs can lead to increased bone mineral density locally [39,40] and greater cartilage loss at the same site [5]. A local effect would thus be consistent with the existing literature.

Higher BMI and strenuous activity were found to predict BML change. Obesity is a strong risk factor for OA [41], and prior studies have also reported a cross-sectional association between BMI and BML prevalence and severity [42]. However, a 36-month follow-up found no association between BML progression and BMI [42]. Similarly, high-intensity physical activity has been shown to increase the risk of OA [43,44]. However these findings need to be balanced against the strong evidence demonstrating that physical activity improves symptoms and physical function in OA [45]. A recent longitudinal study by our group reported that physical activity measured by steps per day was deleteriously associated with BML change [23]. Whilst we found a significant association between strenuous physical activity and BML change, we did not find an association between light physical activity and BML change, suggesting intensity of activity may be important. Therefore, whilst physical activity may be good for symptoms, excessive physical activity may be detrimental to knee structure. Randomized controlled trials evaluating the effect of physical activity on a sensitive measure of knee structure, such as MRI, are needed to gain a better understanding of this relationship.

Our study has several potential limitations. Firstly, slightly different MRI protocols were used at visit two and visit three. Due to the long follow-up period, the protocol at visit three had slightly different parameters – namely a smaller slice thickness. This means that a greater number of small BMLs might have been picked up at visit three; the rate of incident BMLs over 8 years may therefore not be as high as our study indicates. There may also have been an overestimate of BML change, which would mean that the true magnitude of the association between BML change and change in pain is stronger.

Secondly, due to the long follow-up period, a significant proportion of our subjects were lost to follow-up. However, there were no significant differences in pain scores and demographics between those lost to follow-up and participants in this study, suggesting this bias was not systematic.

Thirdly, BML area was measured by taking the slice with the greatest BML size at a particular site. This is a surrogate measure of volume and may overestimate shallow, flat lesions. However, this method of BML measurement has proven to be sensitive to change in a recent clinical trial [46].

Fourthly, the interslice gap was 0.5 to 1.0 mm, which means that small BMLs might have been missed if they lay completely within the interslice gap, which seems unlikely.

Fifthly, we used a subjective measure of physical activity to assess the amount of light and strenuous activity that participants undertook. We also did not differentiate between different modes of physical activity such as weight-bearing and nonweight-bearing exercise, and did not specifically ask about strenuous incidental physical activity (for example, occupational or household activity). It is also important to note that only current physical activity data were captured over a 14-day period as opposed to a longitudinal measure of physical activity. However, the significant correlation between strenuous activity and BML change is consistent with pedometer-derived physical activity.

Sixthly, we did not assess analgesia such as paracetamol or nonsteroidal anti-inflammatory drugs or knee malalignment, which could have been potential confounders.

Lastly, ROA was assessed at baseline and not at visit two. This difference in timing may result in a slight underestimate in the prevalence of ROA at visit two, which may influence the borderline results.

## Conclusion

In this midlife cohort, the proportion of BMLs increasing in size was similar to those decreasing in size, with the majority remaining stable. Change in BMLs can be predicted by lifestyle factors, namely BMI and strenuous activity. An increase in BML size or a new BML resulted in an increase in pain especially in males and those with a family history of OA.

## Abbreviations

BMI: body mass index; BML: bone marrow lesion; LSC: least significant criterion; MRI: magnetic resonance imaging; OA: osteoarthritis; ROA: radiographic osteoarthritis.

## Competing interests

The authors declare that they have no competing interests.

## Authors’ contributions

YCF and HIK are co-first authors of this article, and were responsible for data management and cleaning, data analysis, and manuscript writing. DA contributed to data collection, data analysis, and manuscript writing. LB contributed to data analysis and manuscript revision. CD designed and carried out the study planning, participated in data analysis, and revised the manuscript. FC designed and carried out the study planning, participated in data analysis, and revised the manuscript. GJ designed and carried out the study planning, participated in data analysis, and revised the manuscript. All authors read and approved the final manuscript.
